# An iliac arterial pseudoaneurysm diagnosed 40 years after suffering blunt trauma

**DOI:** 10.1186/s40792-017-0315-1

**Published:** 2017-03-01

**Authors:** Atsushi Guntani, Eisuke Kawakubo, Shinsuke Mii

**Affiliations:** Department of Vascular Surgery, Saiseikai Yahata General Hospital, 5-9-27 Haruno-machi, Yahatahigashi-ku, Kitakyushu, 805-8527 Japan

**Keywords:** Iliac arterial pseudoaneurysm, Blunt trauma, Chronic course

## Abstract

**Background:**

A chronic iliac arterial pseudoaneurysm caused by blunt trauma is very rare.

**Case presentation:**

The patient had gunshot wound in the right thigh and blunt trauma 40 years earlier. An abdominal computed tomography revealed the presence of a right iliac arterial pseudoaneurysm, and we successfully treated the pseudoaneurysm by resection and in situ reconstruction with a bifurcated vascular prosthesis.

**Conclusions:**

We herein present a rare case of a pseudoaneurysm of the right iliac artery diagnosed decades after blunt trauma.

## Background

Pseudoaneurysms of the iliac artery are rare and are typically caused by infection, blunt trauma, or surgical procedures. The clinical signs and symptoms may appear within a few days of the event [1–4]. We herein report a rare case of a pseudoaneurysm of the right iliac artery that was diagnosed decades after blunt trauma.

## Case presentation

**Fig. 1:**
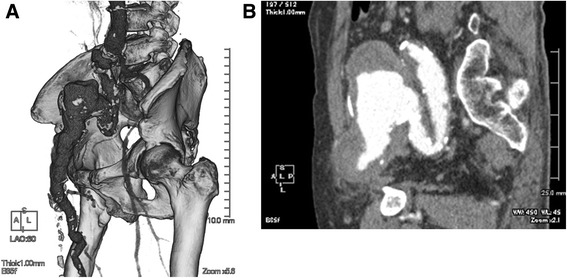
Preoperative contrast-enhanced abdominal computed tomography revealed the presence of an irregular aneurysm from the right common iliac artery to the right common femoral artery, the maximum diameter of which was 50 mm (**a** left anterior oblique view in three-dimensional computed tomography; **b** sagittal view)

**Fig. 2:**
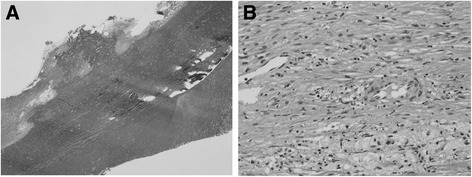
A pathological examination showed that the arterial wall structure had disappeared and been replaced by fibrous tissue with hyalin, hemosiderin, and macrophage infiltration (**a** elastica van Gieson staining, low-magnification; **b** hematoxylin and eosin staining, high-magnification)

**Fig. 3:**
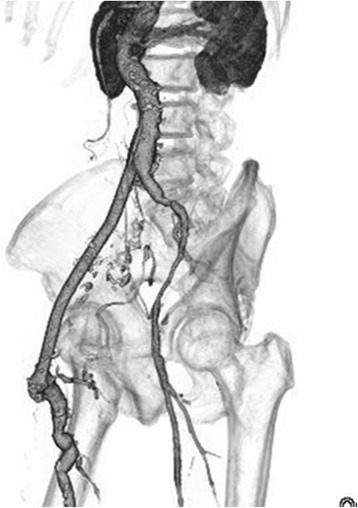
Follow-up contrast-enhanced computed tomography, which was performed 6 months after the surgery, revealed that no recurrence of any other infection or inflammation

A 69-year-old male patient was admitted for right hip pain. The patient had been healthy until he developed a cardiogenic cerebral infarction in 5 years prior to this presentation. Although the details were unclear, the patient had suffered a gunshot wound to the right thigh and the superficial femoral artery had been ligated to achieve hemostasis 40 years previously. He had also been thrown from a bridge of approximately 40 m in height. At that time, he injured his waist and back, however, he successfully recovered while being conservatively treated. The relationship between these traumatic events and the current event was unknown; however, he had been aware of a pulsatile mass in his right groin for at least 20 years. Subsequent contrast-enhanced abdominal computed tomography revealed the presence of an irregular aneurysm from the right common iliac artery to the right common femoral artery, the maximum diameter of which was 50 mm (Fig. 1a, b). Since the patient had no history of connective tissue disorders or vasculitis, and a blood analysis revealed no inflammatory findings, we clinically diagnosed the patient with a right iliac arterial pseudoaneurysm, which was thought to have been caused by an old infection or past trauma. We performed surgery to resect the aneurysm and performed in situ reconstruction with a bifurcated vascular prosthesis (Hemashield 16 × 8 mm; MAQUET Holding B.V. & Co. KG, Rastatt, Germany). Since the right common femoral artery was enlarged and showed severe adhesion to the surrounding tissues due to previous surgery for the gunshot wound, we decided to use the right deep femoral artery for peripheral anastomosis. On entering the abdominal cavity, the right iliac arterial aneurysm was irregular, with a whitish surface, and it adhered to the small intestines. These findings were suggestive of chronic inflammation. According to the usual procedure, the prosthetic bifurcated graft was anastomosed to the transected aorta, distal of the inferior mesenteric artery, and the left limb of the graft was anastomosed to the left common iliac artery. When the right limb of the graft was introduced to the right inguinal region through the incised common iliac arterial pseudoaneurysm, we found that the right external iliac arterial aneurysm had penetrated the right common iliac arterial aneurysm. Part of the aneurysmal wall was excised and submitted for culturing and microscopic examination. The reconstructed prosthetic bifurcated graft was wrapped with the remaining aneurysmal wall and omentum. No bacteria were detected in the resected aneurysmal wall tissue, and a pathological examination showed that the arterial wall structure had disappeared and been replaced by fibrous tissue with hyalin, hemosiderin, and macrophage infiltration (Fig. 2a, b). No recurrence of any other infection or inflammation was observed during the 1-year follow-up period (Fig. 3).

### Discussion

Pseudoaneurysms are often caused by infection or trauma. The number of iatrogenic cases has increased in recent years; however, the patient in our case had never undergone laparotomy or interventional procedures in the past. Furthermore, symptoms due to aneurysms are likely to appear early after trauma, and the rupture of a pseudoaneurysm represents a life threatening event [1, 2]. Few cases describe symptoms that appeared several years later [3, 4]. Our case had experienced traumatic events 40 years previously and had been aware of a pulsatile mass in his right groin for at least 20 years. Based on this evidence, it is assumed that the pseudoaneurysm of the iliac artery increased chronically. Although stent grafts have been reported to be effective for the treatment of pseudoaneurysms [5, 6], we decided to perform open surgery because the aneurysm extended to the common femoral artery, which meant that there was not enough landing zone for stent graft.

No pus or ascites was observed during surgery, and there were no findings of infection from cultures or resected specimens. Additionally, the iliac arterial aneurysms had penetrated each other and a pathological examination showed that the arterial wall structure had disappeared. We considered that the iliac arterial aneurysm had been caused by past trauma and that it might have been sealed by surrounding tissue in the retroperitoneal cavity and gradually increased in size. Thereafter, the pseudoaneurysm gradually expanded and became associated with local infection and inflammation from the common iliac artery to the common femoral artery. The possibility of infectious aneurysms was ruled out based on the lack of inflammatory findings and the negativity of the blood culture. Indeed, no apparent infections were observed intraoperatively or in an examination of the excised specimens. As a result, the postoperative course was uneventful, and no recurrence of any other infection or inflammation was observed during the 1-year follow-up period.

## Conclusions

We herein presented a rare case of the successful treatment of a pseudoaneurysm of the right iliac artery that was diagnosed decades after blunt trauma. Since pseudoaneurysms may occur even a long period after a traumatic event, computed tomography should be used to make a precise diagnosis. Treatment should be administered soon after the diagnosis because of the high rate of mortality associated with the rupture of these lesions.
